# NF-*κ*B mediates proteolysis-inducing factor induced protein degradation and expression of the ubiquitin–proteasome system in skeletal muscle

**DOI:** 10.1038/sj.bjc.6602402

**Published:** 2005-02-15

**Authors:** S M Wyke, M J Tisdale

**Affiliations:** 1 Pharmaceutical Sciences Research Institute, Aston University, Birmingham, B4 7ET, UK

**Keywords:** protein degradation, proteolysis-inducing factor (PIF), NF-*κ*B, proteasome proteolysis

## Abstract

Loss of skeletal muscle in cancer cachexia has a negative effect on both morbidity and mortality. The role of nuclear factor-*κ*B (NF-*κ*B) in regulating muscle protein degradation and expression of the ubiquitin–proteasome proteolytic pathway in response to a tumour cachectic factor, proteolysis-inducing factor (PIF), has been studied by creating stable, transdominant-negative, muscle cell lines. Murine C_2_C_12_ myoblasts were transfected with plasmids with a CMV promoter that had mutations at the serine phosphorylation sites required for degradation of I-*κ*B*α*, an NF-*κ*B inhibitory protein, and allowed to differentiate into myotubes. Proteolysis-inducing factor induced degradation of I-*κ*B*α*, nuclear accumulation of NF-*κ*B and an increase in luciferase reporter gene activity in myotubes containing wild-type, but not mutant, I-*κ*B*α* proteins. Proteolysis-inducing factor also induced total protein degradation and loss of the myofibrillar protein myosin in myotubes containing wild-type, but not mutant, plasmids at the same concentrations as those causing activation of NF-*κ*B. Proteolysis-inducing factor also induced increased expression of the ubiquitin–proteasome pathway, as determined by ‘chymotrypsin-like’ enzyme activity, the predominant proteolytic activity of the *β*-subunits of the proteasome, protein expression of 20S *α*-subunits and the 19S subunits MSS1 and p42, as well as the ubiquitin conjugating enzyme, E2_14*k*_, in cells containing wild-type, but not mutant, I-*κ*B*α*. The ability of mutant I-*κ*B*α* to inhibit PIF-induced protein degradation, as well as expression of the ubiquitin–proteasome pathway, confirms that both of these responses depend on initiation of transcription by NF-*κ*B.

Loss of skeletal muscle results in weakness, immobility and finally death of the cancer patient. Depletion of myofibrillar proteins in muscle results from a decreased protein synthesis (Lundholm *et al*, 1976) combined with an increased protein breakdown (Lundholm *et al*, 1982). The increase in protein breakdown is possibly the most important component of muscle cachexia, since anabolic stimuli, such as nutritional supplementation, fail to reverse the muscle wasting (Evans *et al*, 1985).

The major catabolic pathway involved in degradation of myofibrillar proteins in a range of catabolic conditions, including cancer cachexia, is the ubiquitin–proteasome proteolytic system (Attaix *et al*, 1998). In this process, protein substrates are conjugated with a polyubiquitin chain, which enables them to be recognised for degradation by the proteasome, a multi-subunit complex containing a range of proteolytic enzymes. The main mediators known to influence expression of polyubiquitin genes and proteasomal subunits are glucocorticoids (Wang *et al*, 1998), cytokines such as tumour necrosis factor-*α* (TNF-*α*) (Li *et al*, 1998) and proteolysis-inducing factor (PIF) (Lorite *et al*, 2001), a sulphated glycoprotein produced by cachexia-inducing murine and human tumours (Todorov *et al*, 1996), which specifically induces degradation of skeletal muscle (Lorite *et al*, 1998).

Several studies have investigated the role of the nuclear transcription factor, nuclear factor-*κ*B (NF-*κ*B), in induction of proteasome gene expression. The human proteasome C3 subunit promoter contains elements homologous to the consensus NF-*κ*B-binding site (Du *et al*, 2000), suggesting that NF-*κ*B may be involved in gene transcription. However, the mechanism by which this occurs appears to be diametrically opposite for glucocorticoids (Du *et al*, 2000) and TNF-*α* (Li and Reid, 2000). Thus, glucocorticoids stimulate proteasome expression (at least the C3 subunit) by antagonising the interaction of NF-*κ*B with its response element in the proteasome promoter region. Glucocorticoids also induce gene transcription and protein synthesis of the NF-*κ*B inhibitor, I*κ*B, and inhibit the expression of cytokines (Almawi and Melemedjian, 2002). In contrast, induction of protein degradation by TNF-*α*, which is also mediated through the ubiquitin–proteasome proteolytic pathway (Li *et al*, 1998), appears to be mediated through proteasomal degradation of I*κ*B*α* and translocation of NF-*κ*B to the nucleus (Li and Reid, 2000). We have also recently shown that both PIF (Whitehouse and Tisdale, 2003) and 15(S)-hydroxyeicosatetraenoic acid (15(S)-HETE), an intracellular signal for the increased protein degradation induced by PIF (Whitehouse *et al*, 2003), cause degradation of I*κ*B*α* and nuclear accumulation of NF-*κ*B, associated with an increased proteasome expression. Moreover, attenuation of this process, either by the polyunsaturated fatty acid, eicosapentaenoic acid (EPA), or the NF-*κ*B inhibitor peptide, SN50, also attenuated the PIF-induced increase in proteasome expression, suggesting that NF-*κ*B may act as a transcription factor in the PIF-induced increase in proteasome expression.

To investigate the role of NF-*κ*B in PIF-induced protein degradation and proteasome expression, murine myoblasts have been transfected with viral plasmid constructs that induce overexpression of mutant I*κ*B*α* proteins, that are insensitive to degradation via the ubiquitin–proteasome pathway, and, which, selectively inhibit NF-*κ*B activation (Li and Reid, 2000). The effect of PIF on protein degradation and expression of regulatory components of the ubiquitin–proteasome pathway has been compared in fully differentiated forms of these cells containing mutant I-*κ*B*α*, with those transfected with the empty viral vector.

## MATERIALS AND METHODS

### Materials

L-[2,6^3^H] phenylalanine (sp.act.2.00 TBq mmol^−1^) was purchased from Amersham International (Bucks, UK). Foetal calf serum (FCS), horse serum (HS), Dulbecco's modified Eagle's medium (DMEM), OPTI-MEM1 reduced medium and lipofectamine were purchased from Life Technologies (Paisley, Scotland). Mouse monoclonal antibodies to 20S proteasome *α* subunits, MSS1 and p42 were purchased from Affiniti Research Products, (Exeter, UK), while mouse monoclonal antibody to myosin heavy chain was from Novocastra (Newcastle, UK). Rabbit polyclonal antisera to murine I-*κ*B*α* was from Calbiochem (Herts, UK), that to mouse actin was from Sigma Aldridge (Dorset, UK) and that to ubiquitin conjugating enzyme (E2_14k_) was a gift from Dr Simon Wing, McGill University (Montreal, Canada). Peroxidase-conjugated rabbit anti-mouse antibody and peroxidase-conjugated goat anti-rabbit antibody were purchased from Dako Ltd (Cambridge, UK), Hybond A nitrocellulose membranes were from Amersham International (Bucks, UK). Electrophoretic-mobility shift (EMSA) gel shift assay kits were from Panomics (CA, USA). *Escherichia coli* DH5*α* cells were from Gibco BRL (Paisley, Scotland). Plasmid constructs were under the control of the cytomegalovirus (CMV) promoter and were gifts from Dr Yi-Ping Li (Baylor College of Medicine, Houston, TX, USA). These consisted of empty pCMV4 vector used for the control cell line I-*κ*B*α*ΔN (truncation of amino acids 1–36) and I-*κ*B*α* S32/A36 (point mutations of Ser^32^ and Ser^36^ to alanine). Plasmid DNA was purified using WIZARD Magnesil™ purification kit (Promega, Southampton, UK) according to the manufacturer's protocol. Primers for PCR analysis were purchased from MWG Biotech (Ebersberg, Germany). Gene Juice for transfection studies was obtained from Gene Flow (Staffordshire, UK). The luciferase reporter assay kit was purchased from BD Biosciences Clontech, Oxford, UK. The kinetic-QCL endotoxin assay kit was from Bio Whittaker, MD, USA.

### Purification of PIF

Proteolysis-including factor was purified from solid MAC16 tumours excised from mice with a weight loss between 20 and 25% as described previously (Todorov *et al*, 1996; Whitehouse and Tisdale, 2003). Tumours were homogenised in 10 mM Tris–HCl, pH 8.0, containing 0.5 mM phenylmethylsulphonyl fluoride, 0.5 mM EGTA and 1 mM dithiothreitol at a concentration of 5 ml g^−1^ tumour. The supernatant obtained after addition of ammonium sulphate (40% w v^−1^) was subjected to affinity chromatography using anti-PIF monoclonal antibody coupled to a solid matrix. The immunogenic fractions were concentrated and used for further studies. The purity of the PIF was confirmed by polyacrylamide gel electrophoresis and immunoblotting. This showed a band for PIF at Mr 24 000, sometimes accompanied by an albumin-bound band at Mr 69 000. No other bands were apparent. The endotoxin content of the preparation was below the level of detection.

### Production of transformed colonies

Transformation of plasmid DNA into *E. coli* was achieved using DH5*α* cells. Plasmid DNA was serially diluted to 0.015 *μ*g (*μ*l)^−1^ to perform transformations and 5 *μ*l of diluted DNA was added to 70 ml of competent DH5*α* cells in a chilled microcentrifuge tube and mixed before incubating on ice for 30 min. The cells were then heat shocked for 30 s at 37°C and immediately put back on ice for 2 min. LB medium (500 *μ*l) was added and cells were further incubated at 37°C for 40 min. Aliquots (200 *μ*l) of the transformed cells were spread on LB agar plates containing ampicillin and the plates were incubated overnight at 37°C. Controls for the transformation included a positive control of PUC19 and a negative control of DH5*α* alone. PCR analysis was employed to identify transformed colonies using primers directed against the I-*κ*B*α* insert (forward: GCT GTG ATC ACC AAC CAG C; reverse: CTC TGG CAG CAT CTG AAG G) and for plasmid DNA for those containing pCMV4 (forward: GGT CTA TTC GGG AAC CAA G; reverse: CAC ATT CCA CAG AAG CTG C).

### Myogenic cell culture and transfection

The C_2_C_12_ myoblast cell line was grown in DMEM supplemented with 10% FCS plus 1% penicillin and streptomycin under an atmosphere of 10% CO_2_ in air. Stable transfections were carried out on cells at 50–80% confluency using GeneJuice, according to the manufacturer's protocol, and selected by resistance to ampicillin (5 g l^−1^) as described previously (Smith *et al*, 2004). Transfected myoblasts were stimulated to differentiate by replacing the growth medium with DMEM supplemented with 2% HS, when the cells reached confluence. Differentiation was allowed to continue for 3–5 days until myotubes were clearly visible.

### Luciferase reporter gene assay

The assay was performed using the method described by the supplier. In brief, C_2_C_12_ myoblasts containing each I*κ*B*α* insert were seeded in 75 cm^2^ flasks without antibiotics and incubated until 80% confluent. Then, cells were washed twice with OPTI-MEM Reduced Medium, followed by the addition of 6 ml of OPTI-MEM Reduced Medium containing either 15 *μ*g of NF-*κ*B luciferase reporter plasmid or 15 *μ*g of control luciferase plasmid and 45 *μ*l of Lipofectamine reagent. After 24 h incubation, the cells were passaged into six-well plates at 1 × 10^5^ cells per well, allowed to reach confluence, and differentiated into myotubes. Cells were treated with PIF for 1 h at varied concentrations between 0 and 16.8 nM and washed twice in PBS. 1 × cell lysis buffer was added to cells and cell extracts were immediately assayed for luciferase activity using a BioOrbit luminometer 1253 (Turku, Finland). This was the earliest time point at which an increase in luciferase activity was observed.

### Measurement of protein degradation

This was determined as described previously (Whitehouse and Tisdale, 2003) by prelabelling cells for 24 h with L-[2,6^3^H]phenylalanine (0.67 mCi mmole^−1^), followed by extensive washing in PBS and further incubation for 2 h in DMEM without phenol red, until no more radioactivity appeared in the supernatant. Protein degradation was determined by the release of [2,6^3^H]phenylalanine into the medium after 24 h in the presence of various concentrations of PIF together with 2 mM cold phenylalanine to prevent reincorporation of radioactivity in the cells.

### Measurement of proteasome activity

‘Chymotrypsin-like’ enzyme activity was determined fluorimetrically by the method of Orino *et al* (1991) by the release of aminomethyl coumarin (AMC) from the fluorogenic peptide succinyl-LLVY-AMC. This method has been described previously for C_2_C_12_ myotubes (Whitehouse and Tisdale, 2003). Activity was measured in the absence and presence of the specific proteasome inhibitor lactacystin (10 *μ*M). Only lactacystin-suppressible activity was considered to be proteasome specific.

### Western blot analysis

Myotubes were incubated with various concentrations of PIF as depicted in the figure legends, after which the medium was removed and the cells were washed with PBS and scraped from the plastic surface. They were then sonicated at 4°C in 500–2000 *μ*l of 20 mM Tris–HCl, pH 7.5, 2 mM ATP, 5 mM MgCl_2_ and 1 mM dithiothreitol (DTT). Samples of cytosolic protein (5–30 *μ*g), formed by centrifugation at 18 000 **g** for 5 min, were resolved on 12% sodium dodecylsulphate, polyacrylamide gels (SDS/PAGE) and transferred to 0.45 *μ*m nitrocellulose membranes, which had been blocked with 5% Marvel in Tris-buffered saline, pH 7.5, at 4°C overnight. The primary antibodies were used at a dilution of 1 : 1000 except for actin (1 : 100), and the secondary antibodies were also used at a dilution of 1 : 1000. Incubation was for 1 h at room temperature and development was by enhanced chemiluminescence (ECL) (Amersham, UK). Blots were scanned by a densitometer to quantitate differences.

### Electrophoresis mobility shift assay (EMSA)

DNA-binding proteins were extracted from myotubes according to the method of Andrews and Faller (1991), which utilises hypotonic lysis followed by high salt extraction of nuclei. The EMSA-binding assay was carried out using a Panomics EMSA ‘gel shift’ kit according to the manufacturer's instructions.

### Statistical analysis

Differences in means between groups were determined by one-way ANOVA, followed by Tukey's post-test.

## RESULTS

To determine whether NF-*κ*B mediates PIF-induced protein degradation and upregulation of the ubiquitin–proteasome proteolytic pathway in muscle, C_2_C_12_ murine myoblasts were transfected with either of two dominant-negative mutants of I-*κ*B*α*. In I-*κ*B*α* ΔN, the phosphorylation sites required for degradation (Ser^32^ and Ser^36^) are absent (truncation of amino acids 1–36), while in I-*κ*B*α* S32/A36 there are point mutations of Ser^32^ and Ser^36^ to alanine. This prevented ubiquitin conjugation and proteolysis of either protein (Brockman *et al*, 1993; Chen *et al*, 1995). The control cells were transfected with the empty pCMV4 vector. Transfected myoblasts were allowed to differentiate into myotubes for further studies.

**Figure 1:**
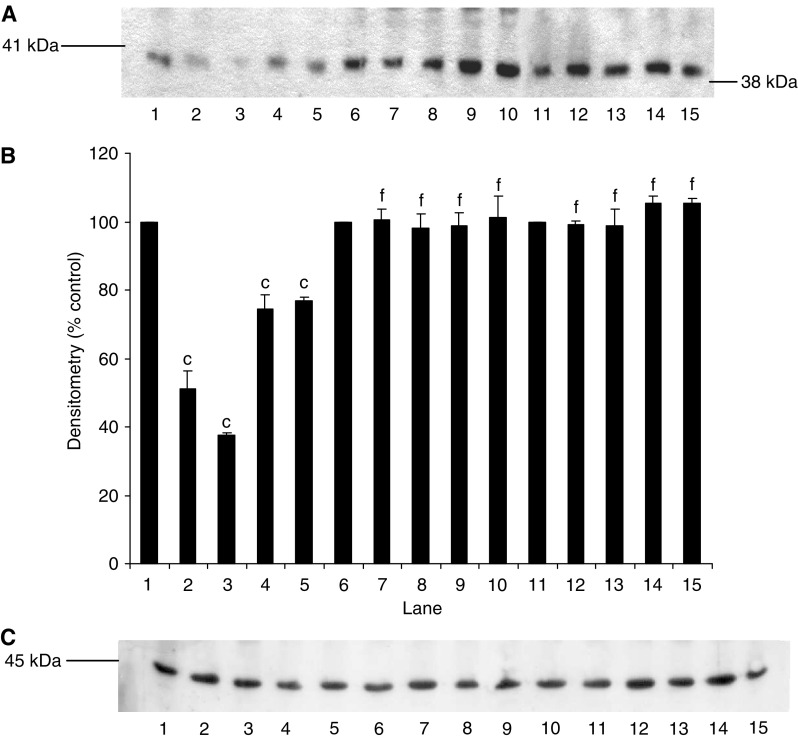
Effect of mutation on degradation of I-*κ*B*α* in the presence of PIF. (**A**) Western blot analysis of I-*κ*B*α* after 30 min incubation with 0 (lanes 1, 6 and 11), 2.1 (lanes 2, 7 and 12), 4.2 (lanes 3, 8 and 13), 10.5 (lanes 4, 9 and 14) and 16.8 (lanes 5, 10 and 15) nM PIF in wild-type cells transfected with pCMV4 (lanes 1–5), I-*κ*B*α*S32/A36 (lanes 6–10) and I-*κ*B*α*ΔN (lanes 11–15). (**B**) Densitometric analysis of the blot shown in (**A**) as the mean±s.e.m. for three separate determinations. Differences from control are indicated as c, *P*<0.001, while differences from wild-type are shown as f, *P*<0.001. (**C**) Western blot of actin showing equal loading of samples in (**A**).

**Figure 2:**
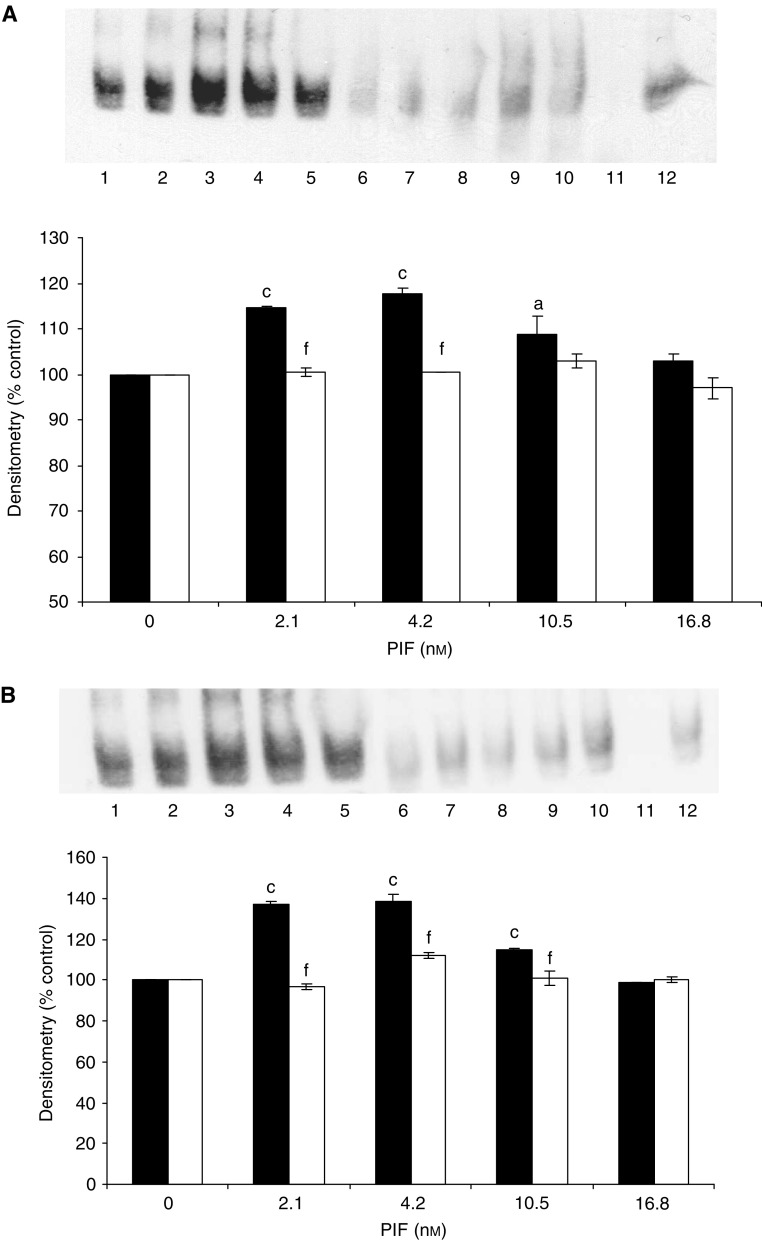
Activation of NF-*κ*B binding to DNA as demonstrated by EMSA. Only the band for bound NF-*κ*B is shown. C_2_C_12_ myotubes were treated with 0 (lanes 1 and 6), 2.1 (lanes 2 and 7), 4.2 (lanes 3 and 8), 10.5 (lanes 4 and 9) and 16.8 (lanes 5 and 10) nM PIF. Binding of NF-*κ*B to nuclear proteins was determined in cells transfected with pCMV4 (lanes 1–5) and I-*κ*B*α* S32/A36 (lanes 6–10) (**A**) and in pCMV4 (lanes 1–5) and I-*κ*B*α*ΔN (lanes 6–10) (**B**). Lane 12 is a positive control for NF-*κ*B (supplied by the manufacturer of the kit), while lane 11 contains the positive control for NF-*κ*B together with a 100-fold excess of unlabelled NF-*κ*B probe. The densitometric analysis of the blots is shown underneath the EMSA. Wild type is shown as solid boxes, while the mutants are shown as open boxes. Figures are means±s.e.m. for three separate determinations. Differences from control are indicated as a, *P*<0.05 and c, *P*<0.001, while differences from wild-type are shown as f, *P*<0.001.

Proteolysis-inducing factor induced a decrease in I-*κ*B*α* (Figure 1, lanes 2–5) and an increase in nuclear accumulation of NF-*κ*B (Figure 2, lanes 2–4) in control myotubes transfected with the pCMV4 vector, which was the same as that previously observed in C_2_C_12_ myotubes (Whitehouse and Tisdale, 2003). In contrast, overexpression of I-*κ*B*α* ΔN or I-*κ*B*α* S32/A36 inhibited degradation of I-*κ*B*α* in the presence of PIF (Figure 1, lanes 6–15), as well as nuclear translocation of NF-*κ*B (Figure 2, lanes 6–10). Myoblasts transfected with these mutant plasmids have previously been shown not to respond to TNF-*α* with nuclear translocation of NF-*κ*B (Li and Reid, 2000), in contrast with those containing the empty vector. Myotubes containing the truncated I-*κ*B*α* (I-*κ*B*α*ΔN) showed a lower molecular weight for I-*κ*B*α* (Figure 1, lanes 11–15) as expected.

**Figure 3:**
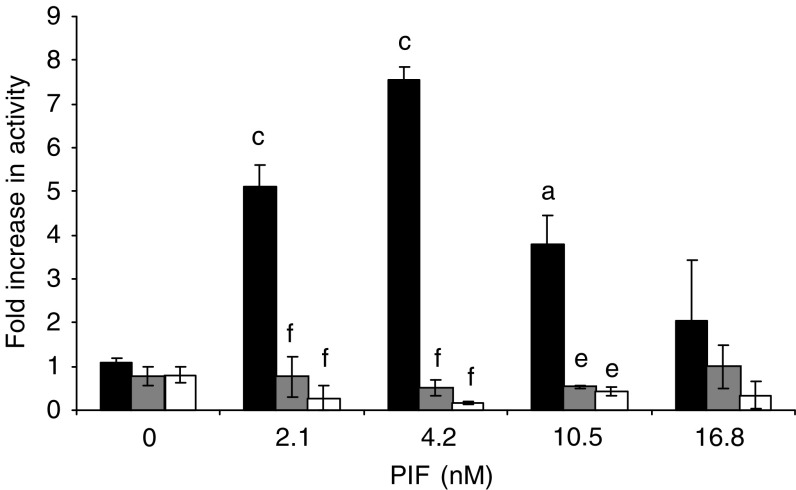
Effect of PIF on the transcriptional activity of NF-*κ*B measured by the luciferase reporter gene assay in myotubes transfected with pCMV4 (▪), S32/A36 () and I-*κ*BΔN (□) after 1 h incubation. Sample luciferase activity was normalised to control luciferase activity. The results shown are mean±s.e.m., where *n*=3. Differences from 0 nM PIF for pCMV4 is shown as a, *P*<0.05 or c, *P*<0.001, while differences from wild-type myotubes are indicated as e, *P*<0.01 or f, *P*<0.001.

To determine whether the increased nuclear translocation of NF-*κ*B was linked to an increased transcriptional activity, myoblasts containing the mutant plasmids or the empty vector were transfected with a plasmid vector with the NF-*κ*B-binding site in the promoter region of the reporter luciferase gene and allowed to differentiate into myotubes. When treated with 2.1–10.5 nM PIF for 1 h, the luciferase activity was between 3.5- and 7.5-fold higher relative to untreated control in myotubes transfected with the empty pCMV4 vector, but was not increased in either of those containing the mutant plasmid (Figure 3). These results confirm that activation of NF-*κ*B by PIF causes an increase in transcriptional activity.

**Figure 4:**
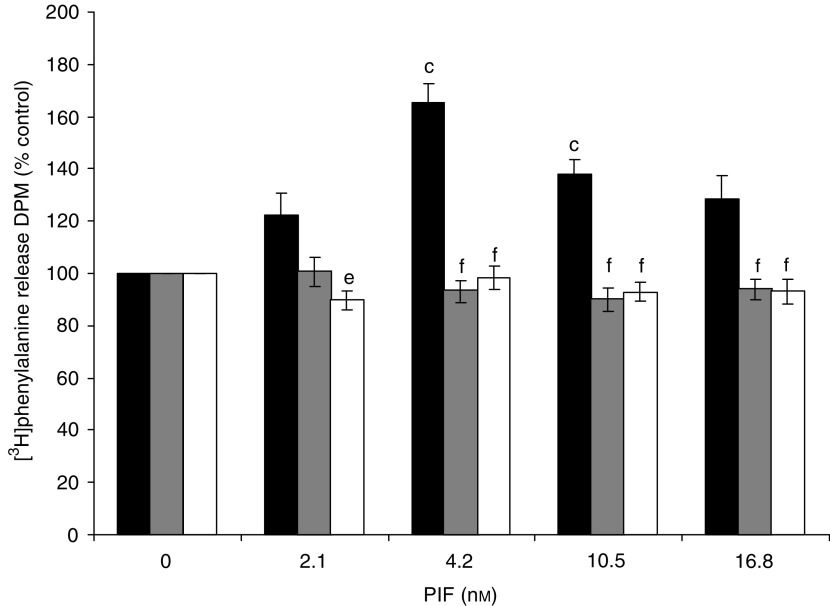
Effect of PIF on total protein degradation in myotubes transfected with pCMV4 (▪), I-*κ*B*α* S32/A36 () and I-*κ*B*α*ΔN (□) over a 24 h period. Results are shown as mean±s.e.m. of one experiment, where *n*=6, and the experiment was repeated twice on different days with similar results. Differences from 0 nM PIF for pCMV4 is shown as c, *P*<0.001, while differences between I-*κ*B*α* S32/A36 and I-*κ*B*α*ΔN and pCMV4 are shown as e, *P*<0.01 and f, *P*<0.001.

**Figure 5:**
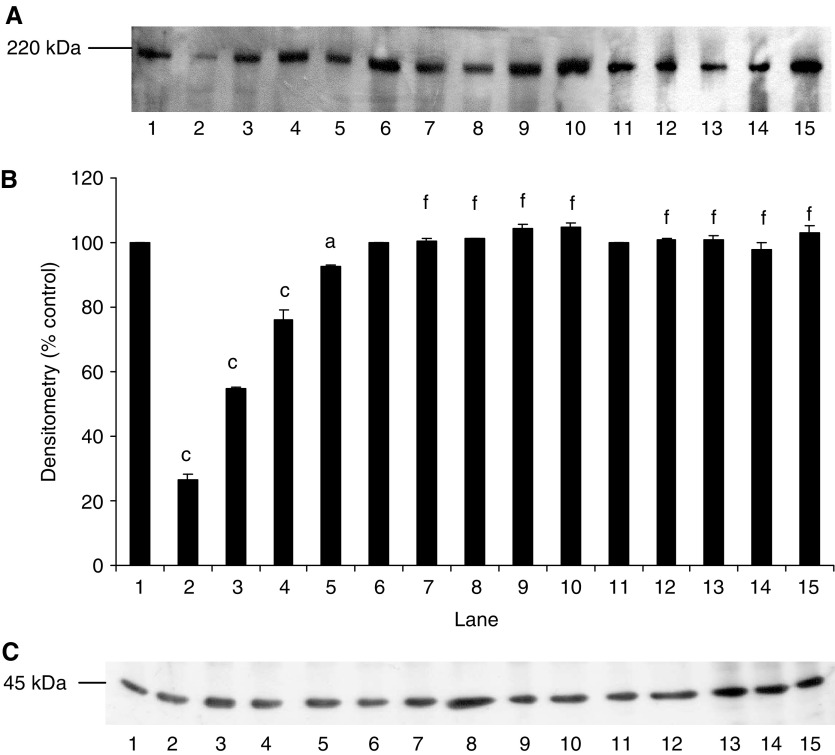
Effect of PIF on myosin content of myotubes containing wild-type and mutant I-*κ*B*α*. (**A**) Wild-type myotubes transfected with pCMV4 (lanes 1–5), I-*κ*B*α* S32/A36 (lanes 6–10) and I-*κ*B*α*ΔN (lanes 11–15) myotubes were incubated with 0 (lanes 1, 6 and 11), 2.1 (lanes 2, 7 and 12), 4.2 (lanes 3, 8 and 13), 10.5 (lanes 4, 9 and 14) or 16.8 (lanes 5, 10 and 15) nM PIF for 24 h and myosin content was determined by Western blotting. (**B**) Densitometric analysis of the blot shown in (**A**). Figures are mean±s.e.m. of three separate determinations. Differences from 0 nM PIF are shown as a, *P*<0.05 and c, *P*<0.001, while differences from wild-type myotubes are indicated as f, *P*<0.001. (**C**) Western blot of actin showing equal loading in (**A**).

The effect of PIF on protein degradation in wild-type and mutant cell lines is shown in Figure 4. Proteolysis-inducing factor produced a significant increase in total protein loss, as measured by [^3^H] phenylalanine release, over the concentration range 2–16.8 nM, in wild-type, but not in mutant cells. The effect was seen over the same concentration range as that inducing I-*κ*B*α* degradation (Figure 1, lanes 2–5) and nuclear accumulation of NF-*κ*B (Figure 2, lanes 2–4) and the increase in luciferase reporter gene activity (Figure 3). The effect in wild-type cells was similar to that previously reported for nontransfected C_2_C_12_ myotubes (Gomes-Marcondes *et al*, 2003). Proteolysis-inducing factor also produced a decrease in the concentrations of the myofibrillar protein myosin in myotubes transfected with the wild-type pCMV4 vector, which was significant at all concentrations of PIF between 2 and 16.8 nM (Figure 5, lanes 1–5). A decrease in myosin was not seen in myotubes that overexpressed either I-*κ*B*α* mutant (Figure 5, lanes 6–15). As previously reported (Acharyya *et al*, 2004) in cachectic mice bearing the colon 26 tumour and in myotubes treated with TNF-*α* and interferon-*γ*, PIF induced selective loss of myosin, while actin levels remained unchanged (Figure 5). This may be due to selective degradation of myosin by the ubiquitin–proteasome pathway. The ability of mutant I-*κ*B*α* to inhibit PIF-induced protein loss indicates that this response depends on NF-*κ*B signalling.

**Figure 6:**
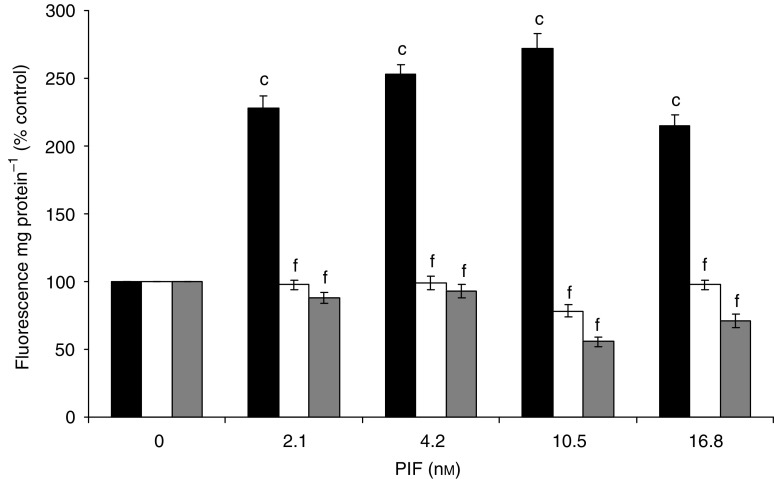
The effect of mutation of I-*κ*B*α* on the chymotrypsin-like enzyme activity in murine myotubes after treatment with PIF. pCMV4 myotubes (▪); I-*κ*B*α* S32/A36 (□) and I-*κ*B*α*ΔN() were treated with the indicated concentrations of PIF for 24 h and the ‘chymotrypsin-like’ enzyme activity was determined fluorimetrically as described in Materials and Methods. Differences from 0 nM PIF for pCMV4 are indicated as c, *P*<0.001, while differences from wild-type myotubes are indicated as f, *P*<0.001.

**Figure 7:**
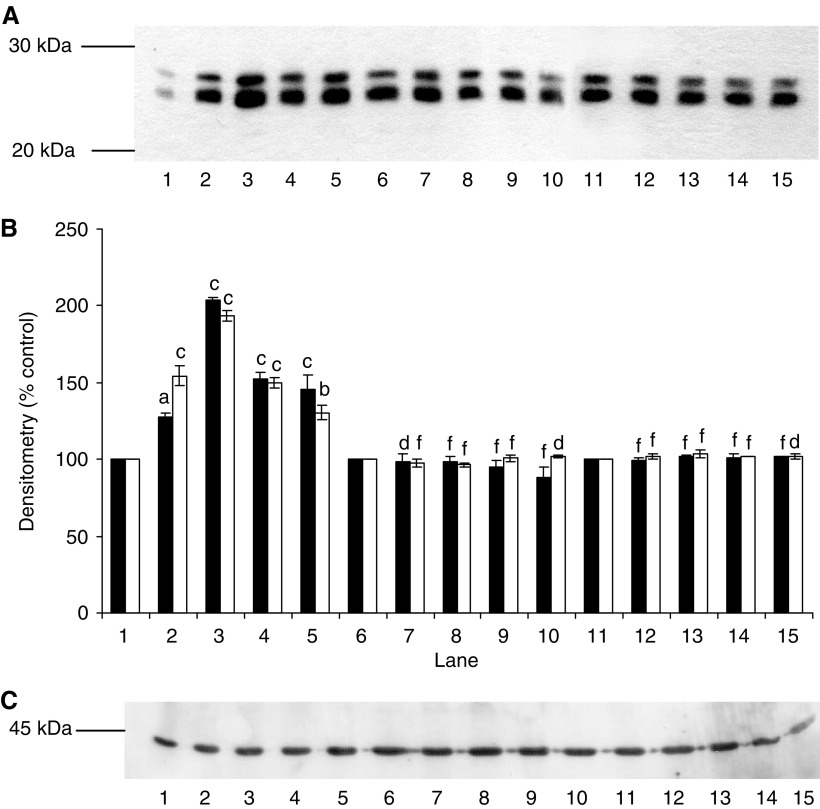
(**A**) Effect of PIF on 20S proteasome *α*-subunit expression in myotubes transfected with pCMV4 (lanes 1–5), I-*κ*B*α* S32/A36 (lanes 6–10) and I-*κ*B*α*ΔN (lanes 11–15) plasmids. Myotubes were incubated for 24 h with 0 (lanes 1, 6 and 11), 2.1 (lanes 2, 7 and 12), 4.2 (lanes 3, 8 and 13), 10.5 (lanes 4, 9 and 14) and 16.8 (lanes 5, 10 and 15) nM PIF and proteasome expression was determined by Western blotting of 5 *μ*g of cytosolic protein. (**B**) Densitometric analysis of three replicate blots as shown in (**A**) ▪ band 1; □ band 2. Differences from 0 nM PIF are indicated as a, *P*<0.05, b, *P*<0.01 and c, *P*<0.001, while differences from wild-type myotubes are indicated as d, *P*<0.05 and f, *P*<0.001. (**C**) Western blot of actin from the blot shown in (**A**).

**Figure 8:**
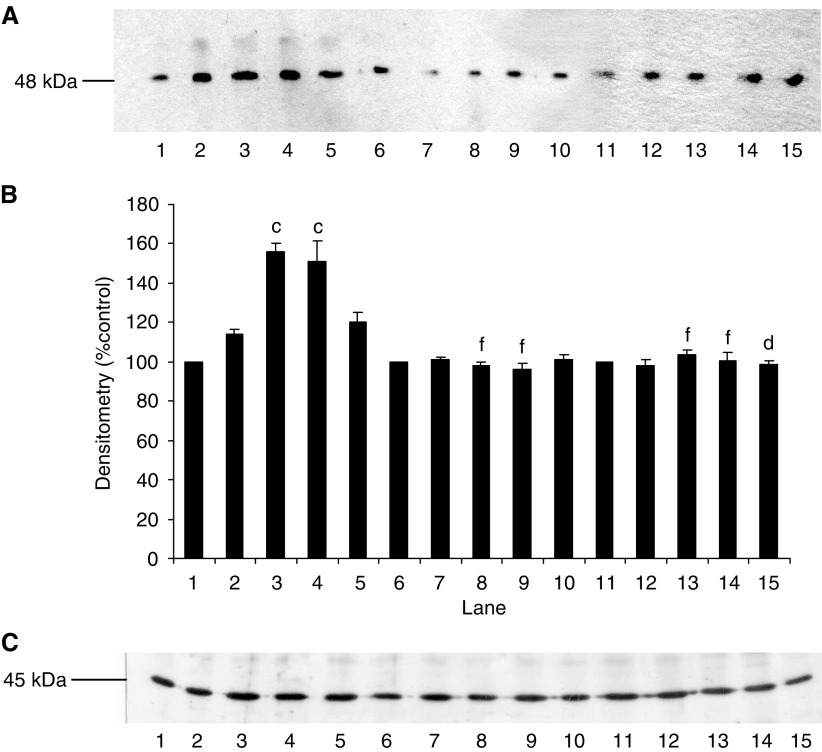
(**A**) Effect of PIF on MSS1 expression in myotubes transfected with wild type (lanes 1–5), I-*κ*B*α* S32/A36 (lanes 6–10) and I-*κ*B*α*ΔN (lanes 11–15) plasmids. Myotubes were incubated for 24 h with 0 (lanes 1, 6 and 11), 2.1 (lanes 2, 7 and 12), 4.2 (lanes 3, 8 and 13), 10.5 (lanes 4, 9 and 14) and 16.8 (lanes 5, 10 and 15) nM PIF and MSS1 expression was determined by Western blotting of 5 *μ*g of cytosolic protein. (**B**) Densitometric analysis of three replicate blots shown in (**A**). Differences from 0 nM PIF are indicated as c, *P*<0.001, while differences from wild-type controls are shown as d, *P*<0.05 and f, *P*<0.001. (**C**) Western blot of actin from the blot shown in (**A**).

**Figure 9:**
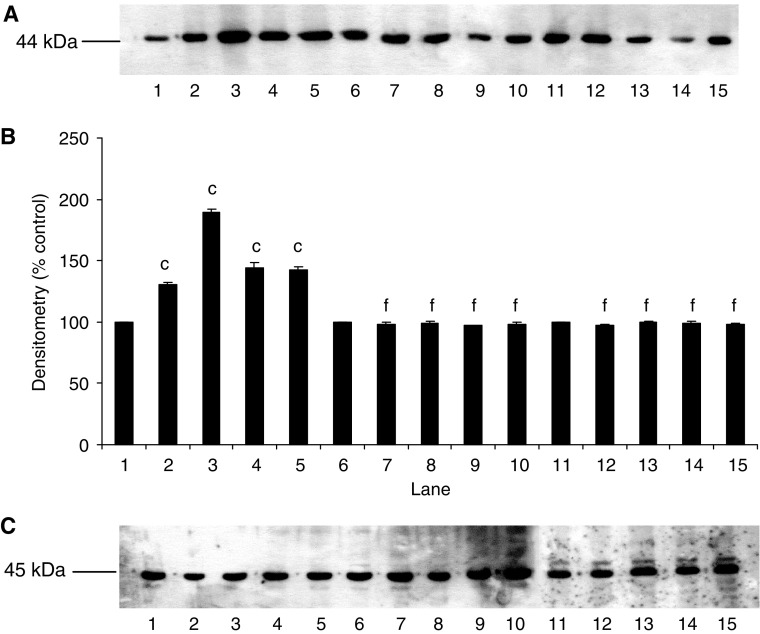
(**A**) Effect of PIF on p42 expression in myotubes transfected with pCMV4 (lanes 1–5), I-*κ*B*α* S32/A36 (lanes 6–10) and I-*κ*B*α*ΔN (lanes 11–15) plasmids. Myotubes were incubated for 24 h with 0 (lanes 1, 6 and 11), 2.1 (lanes 2, 7 and 12), 4.2 (lanes 3, 8 and 13), 10.5 (lanes 4, 9 and 14) and 16.8 (lanes 5, 10 and 15) nM PIF and p42 expression was determined by western blotting. (**B**) Densitometric analysis of three replicate blots shown in (**A**). Differences from 0 nM PIF are shown as c, *P*<0.001, while differences from wild-type controls are shown as f, *P*<0.001. (**C**) Western blot of actin from the blot shown in (**A**).

**Figure 10:**
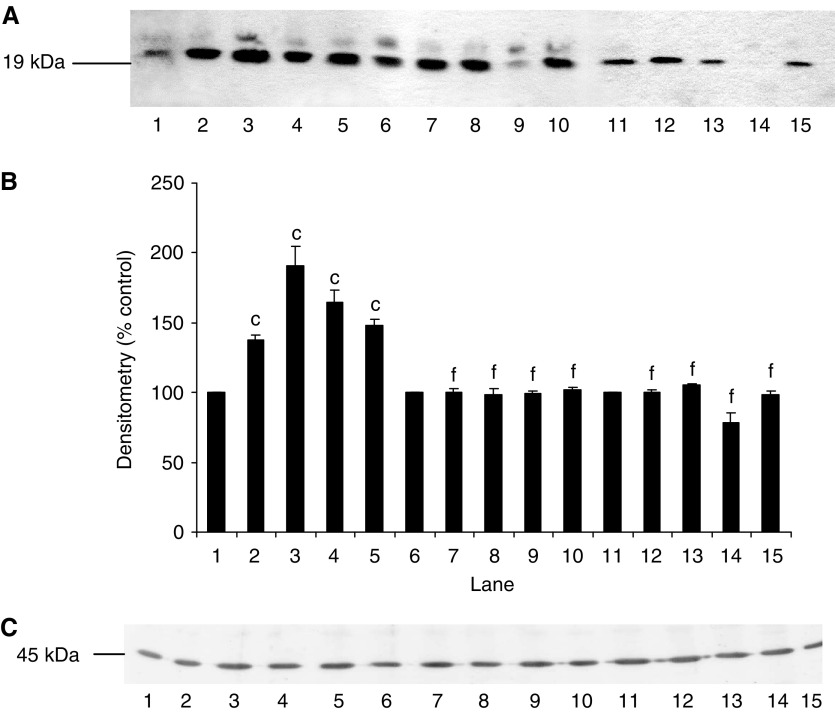
Effect of PIF on E2_14*k*_ expression in myotubes transfected with pCMV4 (lanes 1–5), I-*κ*B*α* S32/A36 (lanes 6–10) and I-*κ*B*α*ΔN (lanes 11–15) plasmids. Myotubes were incubated for 24 h with 0 (lanes 1, 6 and 11), 2.1 (lanes 2, 7 and 12), 4.2 (lanes 3, 8 and 13), 10.5 (lanes 4, 9 and 14) and 16.8 (lanes 5, 10 and 15) nM PIF and p42 expression was determined by Western blotting. (**B**) Densitometric analysis of three replicate blots shown in (**A**). Differences from 0 nM PIF are shown as c, *P*<0.001, while differences from wild-type controls are shown as f, *P*<0.001. (**C**) Western blot of actin from the blot shown in (**A**).

We have previously shown (Lorite *et al*, 2001; Gomes-Marcondes *et al*, 2003) that PIF-induced protein degradation was strongly correlated with an increase in expression of key regulatory components of the ubiquitin–proteasome proteolytic pathway. To determine the role of NF-*κ*B in this process, functional proteasome activity was determined by measuring the ‘chymotrypsin-like’ enzyme activity, the major proteolytic activity of the *β*-subunits. Using the fluorogenic substrate succinyl LLVY-MCA, an increase in enzyme activity was seen at concentrations of PIF between 2 and 16.8 nM in myotubes transfected with the control vector pCMV4 (Figure 6), with a bell-shaped dose–response curve as previously reported (Gomes-Marcondes *et al*, 2003). In contrast, myotubes transfected with both mutant constructs showed no increase in ‘chymotrypsin-like’ enzyme activity in the presence of PIF (Figure 6). Expression of proteasome subunits was determined by Western blotting of cellular supernatants. The effect of PIF on expression of 20S *α*-subunits in wild-type and mutant I-*κ*B*α* transfected myotubes is shown in Figure 7. Proteolysis-inducing factor induced a significant increase in 20S *α*-subunit expression at concentrations between 2 and 10.5 nM in wild-type (Figure 7, lanes 1–5), but not mutant cells (Figure 7, lanes 6–15). Proteolysis-inducing factor also increased expression of MSS1, an ATPase subunit of the 19S regulatory complex, in myotubes transfected with control vector, pCMV4 (Figure 8, lanes 1–5), but not in those transfected with either type of mutant I-*κ*B*α* (Figure 8, lanes 6–15). MSS1, appearing as a single band at Mr∼50 000, was increased in pCMV4 myotubes in the same concentration range as that previously reported in untransfected myotubes (Gomes-Marcondes *et al*, 2003). Proteolysis-inducing factor also increased expression of p42, an ATPase subunit of the 19S regulator that promotes ATP-dependent association of the 20S proteasome with the 19S regulator to form the 26S proteasome (Tanahashi *et al*, 1999) in wild-type (Figure 9, lanes 1–5), but not in mutant myotubes (Figure 9, lanes 6–15). The concentrations of PIF producing an increase in p42 were the same as those producing an increase in 20S *α*-subunit expression (Figure 7). Proteolysis-inducing factor also produced an increase in expression of the Mr 14 000 ubiquitin-conjugating enzyme (E2_14*k*_) in wild type (Figure 10, lanes 1–5), but not in myotubes expressing the mutant form of I-*κ*B*α* (Figure 10, lanes 6–15). Previous studies (Gomes-Marcondes *et al*, 2003) have shown E2_14*k*_ expression to parallel that of proteasome subunits. The ability of mutant I-*κ*B*α* to inhibit PIF-induced proteasome expression confirms that this response also depends on initiation of transcription by NF-*κ*B.

## DISCUSSION

Nuclear factor-*κ*B plays an important role in cellular function including immune and inflammatory responses, regulation of cell growth and apoptosis and tumour induction (Karin *et al*, 2002). In this study, we have utilised I-*κ*B*α* mutated at Ser^32^ and Ser^36^ to investigate a role for NF-*κ*B in protein degradation and induction of the ubiquitin–proteasome proteolytic pathway by PIF. Phosphorylation of I-*κ*B*α* at Ser^32^ and Ser^36^ by the I-*κ*B kinase complex (IKK) leads to ubiquitination of I-*κ*B*α* at nearby lysine residues and degradation by the proteasome (Karin, 1999). An alternative pathway has recently been reported (Fan *et al*, 2003) whereby activation of NF-*κ*B occurs through C-Src-mediated tyrosine phosphorylation of I-*κ*B*α* at residue 42, which is capable of activating NF-*κ*B in the absence of ubiquitin-dependent degradation of I-*κ*B*α*. Such a pathway normally occurs in the redox activation of NF-*κ*B. That such a pathway is not operative in C_2_C_12_ myotubes in the presence of PIF is shown by the lack of nuclear accumulation of NF-*κ*B in cells transfected with the I-*κ*B*α* mutant plasmids in the presence of PIF. This confirms that a lack of response in myotubes containing the mutant plasmids means that the effect is mediated through NF-*κ*B.

The present observations indicate that NF-*κ*B activation is involved in PIF-induced degradation of cellular proteins in skeletal muscle. In this respect, PIF appears to be similar to TNF-*α* (Li and Reid, 2000). There are few studies which have looked at NF-*κ*B activation in skeletal muscle under conditions of muscle wasting. However, a recent study (Hunter *et al*, 2002) showed nuclear extracts from the soleus muscle of rats undergoing disuse atrophy to show increased binding of NF-*κ*B oligonucleotides. This complex bound antibody to p50, c-Rel and Bcl-3, but not other NF-*κ*B members, and there was no evidence for the canonical NF-*κ*B pathway involving activation of p65 or I-*κ*B*α*. This pathway, therefore, seems to be different from that induced by PIF.

Disuse atrophy resembles cancer cachexia in that protein degradation is mediated primarily by the ubiquitin–proteasome pathway (De Martino and Ordway, 1998). This suggests that activation of NF-*κ*B may be a common mechanism for increased gene expression of key regulatory components of this pathway by agents such as PIF and TNF-*α* (Li and Reid, 2000). However, this mechanism is opposite to that induced by glucocorticoids, which have been shown (Du *et al*, 2000), at least in L6 muscle cells, to induce expression of the C3 proteasome subunit by antagonising interaction of NF-*κ*B with the response element in the promoter region. Studies on protein degradation during sepsis, which is also mediated through the ubiquitin–proteasome pathway (Tiao *et al*, 1994), showed that NF-*κ*B is increased at early time points (4 h), but decreased at later time points (16 h) (Penner *et al*, 2001). Which of these changes in NF-*κ*B expression are responsible for the increased muscle protein degradation is not known, but the glucocorticoid receptor antagonist RU38486 increases NF-*κ*B, suggesting that the decreased expression is due to glucocorticoids. How apparently opposite changes in NF-*κ*B expression in the same cell type produce the same effect is not known. It is known that NF-*κ*B can have different effects in different cell types. Thus, while in most cell types NF-*κ*B seems to promote cell proliferation and protect against apoptosis, in skin, it appears to oppose proliferation (Dajee *et al*, 2003). It is possible that different NF-*κ*B pathways are affected by glucocorticoids from that of PIF and TNF-*α*. Interestingly, insulin-like growth factor II (IGF-II)-dependent NF-*κ*B activation has been linked to myoblast differentiation (Canicio *et al*, 2001). It is possible that proteolysis of intracellular regulators is required for fusion and that this requires induction of the ubiquitin–proteasome pathway.

This study shows that increased expression of both proteasome subunits and E2_14*k*_ in the presence of PIF is mediated through activation of NF-*κ*B. A recent study (Li *et al*, 2003) shows that TNF-*α* stimulates expression of the ubiquitin carrier protein, UbcH2, a homologue of murine E2_20*k*_ in skeletal muscle, in a process which is regulated at the transcriptional level by NF-*κ*B. UbcH2 appears to act in parallel with E2_14*k*_ targeting a distinct pool of protein for degradation.

Proteolysis-inducing factor has also been shown to activate NF-*κ*B in primary hepatocytes and the human cancer cell line HepG2, resulting in the increased production of interleukin-6 and -8 (IL-6 and IL-8) and C-reactive protein and the decreased production of transferrin (Watchorn *et al*, 2001). There was also an increase in ICAM-1, another NF-*κ*B-inducible gene. These results suggest that the primary effects of PIF on gene expression are mediated through NF-*κ*B. The mechanism by which this occurs has not been completely evaluated, but recent evidence (Smith and Tisdale, 2003) suggests that PIF induces activation of both phospholipases A and C (PLA_2_ and PC-PLC). The former causes the release of arachidonic acid from membrane phospholipids, while diacylglycerol, derived from PC-PLC, has been suggested to provide a positive feedback signal to protein kinase C (PKC) (Smith and Tisdale, 2003). We have shown PKC to be involved in PIF-induced proteasome expression (Smith *et al*, 2004) and PKC may act as a signal for NF-*κ*B activation through phosphorylation and activation of IKK (Fullman *et al*, 1992).

Induction of protein degradation and expression of the ubiquitin–proteasome pathway through activation of NF-*κ*B would provide a mechanism to explain the effect of EPA in attenuating the process in cachectic mice (Whitehouse *et al*, 2001). Eicosapentaenoic acid is also effective clinically in preventing loss of lean body mass in patients with pancreatic carcinoma (Barber *et al*, 1999). We have shown EPA to prevent both degradation of I-*κ*B*α* and nuclear accumulation of NF-*κ*B in murine myotubes in the presence of PIF (Whitehouse and Tisdale, 2003), through attenuation of upstream signalling pathways. Omega-3 polyunsaturated fatty acids have also been shown to inhibit I-*κ*B phosphorylation and NF-*κ*B activation in murine macrophages, although the mechanism is not known (Novak *et al*, 2003). This manuscript provides experimental evidence to support the claim that PIF induces proteasome expression through activation of nuclear binding of NF-*κ*B. Thus, agents capable of inhibiting activation of NF-*κ*B should potentially be capable of inhibiting muscle protein degradation in cancer cachexia if PIF is involved. One such agent, resveratrol, may prove useful for the treatment of muscle wasting in cancer cachexia (Wyke *et al*, 2004).
